# Is there a relationship between factor V Leiden and type 2 diabetes?

**DOI:** 10.1186/1479-5876-7-52

**Published:** 2009-06-26

**Authors:** Corrado Lodigiani, Paola Ferrazzi, Pierpaolo Di Micco, Luca Librè, Stefano Genovese, Ilaria Quaglia, Lidia Luciana Rota

**Affiliations:** 1 Thrombosis Center, Istituto Clinico Humanitas IRCCS, Rozzano (MI), Italy; 2 Unit of Laboratory and Clinical Chemistry, Istituto Clinico Humanitas IRCCS, Rozzano (MI), Italy; 3 Internal Medicine, Fatebenefratelli Hospital of Naples, Italy; 4 Endocrine and Diabetes Unit, Istituto Clinico Humanitas IRCCS, Rozzano (MI), Italy

## Abstract

**Background:**

Diabetes is well known risk factor for thrombotic events. The association between diabetes and venous thromboembolism is still matter of debate. However, during diabetes an acquired thrombophilia is present and is due to the non-enzymatic glycosilation of clotting inhibitors as antithrombin thus leading to hypercoagulable state. A possibile relationship between the presence of FVL gene variant in type 1 or type 2 diabetes has been hypothysed by several reports in the Literature with non-univocal findings.

**Patients and methods:**

Retrospectively we analysed nearly 7000 patients referred to our Thrombosis Center for venous thromboembolism (VTE) then we selected 115 patients underwent to the screening for inherited thrombophilia. All selected patients were divided in 2 groups: the first group (group A) included 64 patients with previous VTE and carriers of factor V Leiden, while the second group (group B) included 51 patients with previous VTE and evetually carriers of thrombophilic defects other than factor V Leiden. Patients of group B acted as control group. 75 g oral glucose tolerance Test (OGTT) recommended by WHO was perfomed to all subjects in the study in order to screen subjects with glucose reduced tolerance or subjects with inducible diabetes. Statistical analysis was performed with STATA 6  with Student t test for unpaired data, with χ^2 ^test or with Fisher exact test where appropriated; differences were considered to be significant if p < 0.05.

**Results:**

We did not find sifferences between glycaemia at baseline and after OGTT between patients with VTE carriers of FVL compared to non-carriers of FVL. We found a relevant increase in the prevalence of IGT and diabetes between patients with VTE carriers of FVL compared to non-carriers of FVL although this increase did not raise statistical significance.

**Discussion:**

our data pointed out an interesting aspect of the linking between FVL gene variant, diabetes and atherothrombosis and other vascular complications, although data on larger population are needed; this aspect may be another relevant topic of research based because also a link between the pathogenesis of venous thrombosis and atherothrombosis has been recently reported in the Literature.

## Background

Diabetes is well known risk factor for thrombotic events [1]. In particular, since Framingham Study has been published diabetes is recognised as one of the more common risk factor for atherothrombosis [2]. Both type 1 diabetes (i.e. insulin dependent) and type 2 diabetes (i.e. not-insulin dependent) are associated to vascular events, in particular if glycated haemoglobin is higher than 7.0% [3]. Actually, in fact, vascular complications of diabetes represent the more common cause of morbidity and mortality of diabetic patients [4,5]. On the other sides the association between diabetes and venous thromboembolism (VTE) is still matter of debate [6,7]. Some Author did not find an association between diabetes and VTE [6], but recently several Authors showed that atherosclerosis and traditional atherosclerotic risk factor as diabetes should be considered also as risk factor for VTE, in particular for idiopathic VTE [8].

Of course, during diabetes an acquired hypercoagulability is present and is due to several factors as to the non-enzymatic glycosilation of clotting inhibitors as antithrombin thus leading to hypercoagulable state [9,10]. This acquired thrombophilia may be added in any case to a possible inherited thrombophilia if such patients is carrier of such thrombophilic gene variant (e.g. A1691G of factor V and\or prothrombin A20210G) or other thrombotic risk factor.

Factor V Leiden (FVL) is a well known inherited thrombophilic condition both in heterozygosity or in homozygosity. The association between FVL and VTE has been frequently described [11], while the association between FVL and atherothrombosis is still matter of discussion in particular for patients with early onset of vascular atherothrombosis [12,13].

A possibile relationship between the presence of FVL gene variant and type 1 or type 2 diabetes has been hypothysed by several reports in the Literature with non-univocal findings [14-16].

The aim of our retropspective study is to find a possible association between factor V Leiden gene variant and type 2 diabetes in a population of patients with previous VTE.

## Patients and methods

We performed a retrospective analysis of nearly 7000 patients referred to our Thrombosis Center for one or more episode of thrombotic disorders. After a first screening we analysed only patients with previous VTE and in this population, we selected subjects that perfomed the screening for inherited thrombophilia after one or more episodes of VTE; so, we selected 115 patients underwent to the screening for inherited thrombophilia.

### Inclusion criteria

All selected patients were divided in 2 groups: the first group (group A) included 64 patients (33 males and 31 females, mean age 54 ± 9 years) with previous VTE and carriers of factor V Leiden as inherited thrombophilic defect, while the second group (group B) included 51 patients (26 males and 25 females, mean age 51 ± 9 years) with previous VTE and carriers of thrombophilic defects other than factor V Leiden. Patients of group B acted as control group.

### Exclusion criteria

We excluded all patients affected by thrombotic disorders other than VTE, and younger than 40 years and with already personal history of diabetes.

### Factor V Leiden identification

Whole blood samples were collected by venipuncture in order to screen the presence of factor V Leiden gene variant.

DNA was extracted using an automated procedure (MagNA PURE, Roche, Italy). Patients were screened for the G1691A gene variant of factor V Leiden using PCR amplification with specific primers and Light Cycler apparatus (Roche, Milan, Italy).

### Latent diabetes or reduced glucose intolerance identification

75 g oral glucose tolerance Test (OGTT) recommended by WHO was perfomed to all subjects in the study. According to the WHO and National Diabetes Data Group (NDDG) guidelines, we diagnosed diabetes if glycaemia after 2 hours from OGTT was higher than 199 mg\dL and reduced glucose tolerance, if glycaemia after 2 hours from OGTT was higher than 139 mg\dL but lower than 199 mg/dl

### Statistical analysis

Data are expressed as mean ± standard deviation (SD) or as number and percentage where appropriated. Statistical analysis was performed with STATA 6  with Student t test for unpaired data or with χ^2 ^test or with Fisher exact test where appropriated; differences were considered to be significant if p < 0.05.

## Results

We did not find significant difference between glycaemia at baseline and two hours after OGTT between subjects with VTE and FVL compared to control group [93.55 ± 13.57 mg\dl vs 91.44 ± 13.19 mg\dl (p: 0.68, not significant) and 104.90 ± 30.04 mg\dl vs 101.38 ± 37.05 mg\dl respectively; (p 0.47, not significant)] (table 1).

**Table 1:** Glycaemic parameters in subjects with VTE and with or without FVL

Parameters	**Patients with VTE and FVL**	**Patients with VTE without FVL**	**p**
Glycaemia at baseline (mg\dl)	93.55 ± 13.57	91.44 ± 13.19	0.68, ns

OGTT (mg\dl)	104.90 ± 30.04	101.38 ± 37.05	0.47, ns

VTE: venous thromboembolism; FVL: factor V Leiden; OGTT: oral glucose tolerance test

ns: not significant

We found a significant increase of subjects with impaired glucose tolerance in the group A (i.e. patients with previous VTE and carriers of FVL gene variant) compared to controls, although this data did not raise statistical significance (5 patients, 7.81% vs 2 patients, 3.92%, p 0.07, not significant) (table 2).

**Table 2:** Prevalence of diabetes or IGT in subjects with VTE and with or without FVL.

	**Patients with VTE and FVL (n. 64)**	**Patients with VTE and without FVL (n. 51)**	**p**
Normal subjects n (%)	52 (81.25)	46 (90.2)	0.31, ns

IGT n (%)	5 (7.81)	2 (3.92)	0.07, ns

Diabetes n (%)	7 (10.94)	3 (5.88)	0.08, ns

VTE: venous thromboembolism; FVL: factor V Leiden; IGT: impaired glucose tolerance

ns: not significant

Similarly we found an increased number of subjects with diabetes in group A (i.e. patients with previous VTE and carriers of FVL gene variant) compared to controls, although this data did not raise statistical significance (7 patients, 10.94% vs 3 patients, 5.88%, p 0.08, not significant) (table 2).

## Discussion

The association between FVL and VTE is well known [11], while the association between FVL and atherothrombosis is still matter of discussion [12]. On the other hand, the association between diabetes and atherothrombosis is well known [9] while the association between diabetes and VTE has not been recognised by data available in the Literature [7]. However, it is already not known why such patients with diabetes develop more vascular complications both as atherothrombosis (of any district) as VTE and other patients with diabetes do not develop vascular complication. In previous years several research suspected a relationship between diabetes and FVL gene variants both for type 1 or type 2 diabetes but not univocal data were found [14-16]. Krekora et al. in fact suspected also a possible genetic co-segregation for both inherited disorders (i.e. type 2 diabetes and factor V Leiden gene variant) [16].

Our data revealed that there is relationship between latent diabetes in patients carriers of FVL with previous VTE, compared to controls although these data did not raise statistical significance. However, an increase of IGT and diabetes in the group A was found versus group B, so inducing the suspect that a relationship between diabetes and FVL may be looked for in larger population. From a methodological point of view, we may suppose that the number of selected patients should be increased in order to have a more appropriate dimension of the problem and this is actually may represent a study limitation; yet, based on the fact that we performed the study on a retrospective analysis we may speculate that our results are of great interest from a clinical point of view, although at this moment did not raise a statistical significance. Furthermore, from a clinical point of view, in fact, these data may explain better the personal and familial trend to develop thrombotic events of such diabetic community, being type 2 diabetes a disease that show multiple gene-gene interactions.

Moreover, a relevant aspect is related to the recent data present in the Literature that are linking more and more the pathophysiology of arterial and venous thrombosis: actually, in fact, it is not known because during the natural history of type 2 diabetes such diabetic patient present a significant number of atherothrombotic events or venous thrombosis or both type of vascular complications or none of them. Our results underline, in fact, that this clinical aspect could be typical of any community and nor for all type 2 diabetic patients because the inherited trend to develop type 2 diabetes is related to a multivariate gene-gene interaction and gene-enviromental interaction.

## Conclusion

So, our data pointed out again an interesting aspect of the linking between FVL gene variant, diabetes and atherothrombosis or other type of vascular complications, although data on larger population are needed and should be evaluated not only as retrospective analysis.

This aspect, in fact, may be another relevant topic of research based on recent data from the Literature that are frequently linking the pathogenesis of venous thrombosis and atherothrombosis.

## Competing interests

The authors declare that they have no competing interests.

## Authors' contributions

CL and SG selected patients enrolled for the study; PF and IQ performed all laboratory tests; PDM and LL performed scientific up-date; CL and LLR perfomed study design and statistical analysis. All authors read and approved the final manuscript.
